# Musculoskeletal pain in Arctic indigenous and non-indigenous adolescents, prevalence and associations with psychosocial factors: a population-based study

**DOI:** 10.1186/1471-2458-14-617

**Published:** 2014-06-18

**Authors:** Christian Eckhoff, Siv Kvernmo

**Affiliations:** 1 Department of Child and Adolescent Psychiatry, Division of Child and Adolescent Health, University Hospital North Norway, Tromsoe N-9038, Norway; 2 Department of Clinical Medicine, Faculty of Health Sciences, University of Tromsoe; The Artic University of Norway, Tromsoe N-9037, Norway

**Keywords:** Musculoskeletal pain, Adolescents, Psychosomatic, Somatization, Psychosocial, Emotional problems, Nordic, Sami, Indigenous

## Abstract

**Background:**

Pain is common in otherwise healthy adolescents. In recent years widespread musculoskeletal pain, in contrast to single site pain, and associating factors has been emphasized. Musculoskeletal pain has not been examined in Arctic indigenous adolescents. The aim of this study was to explore the prevalence of widespread musculoskeletal pain and its association with psychosocial factors, with emphasis on gender- and ethnic differences (Sami vs. non-Sami), and the influence of pain related functional impairment.

**Methods:**

This is a cross-sectional study based on The Norwegian Arctic Adolescent Health Study; a school-based survey responded by 4,881 10th grade students (RR: 83%) in North Norway, in 2003–2005. 10% were indigenous Sami. Musculoskeletal pain was based on reported pain in the head, shoulder/neck, back and/or arm/knee/leg, measured by the number of pain sites. Linear multiple regression was used for the multivariable analyses.

**Results:**

The prevalence of musculoskeletal pain was high, and significantly higher in females. In total, 22.4% reported 3–4 pain sites. We found a strong association between musculoskeletal pain sites and psychosocial problems, with a higher explained variance in those reporting pain related functional impairment and in females. There were no major differences in the prevalence of musculoskeletal pain in Sami and non-Sami, however the associating factors differed somewhat between the indigenous and non-indigenous group. The final multivariable model, for the total sample, explained 21.2% of the variance of musculoskeletal pain. Anxiety/depression symptoms was the dominant factor associated with musculoskeletal pain followed by negative life events and school-related stress.

**Conclusions:**

Anxiety/depression, negative life events, and school-related stress were the most important factors associated with musculoskeletal pain, especially in those reporting pain related functional impairment. The most important sociocultural aspect is the finding that the indigenous Sami are not worse off.

## Background

Children and adolescents commonly express physical complaints without a clear somatic cause [1]. In adolescents musculoskeletal pain is most prevalent [2-4], showing an increasing trend in the last two decades [5], with females being more troubled [4,5]. An association between adolescent pain reports and pain reports in early adulthood has been found [6]. The high prevalence of physical complaints [2,4,5] are considered as possible manifestations of psychosocial problems [7], and a common way of presenting mental health problems in primary care and pediatric setting [1]. Psychosomatic symptoms often coexist and those reporting pain often report pain from a number of sites [8]. In recent years there has been emphasis on widespread musculoskeletal pain, in contrast to single site pain, showing a strong association with psychosocial problems [9-11]. It is more likely that the influence of psychosocial factors is greater in widespread pain than in localized pain, and more studies on widespread pain in adolescents are needed.

Adolescence may be a stressful period, with debut of mental health problems. Physical complaints are associated with anxiety/depressive symptoms [1,10-13], low self-esteem, poor resilience [14], peer-, parental-, or school problems [15-18]. A dose–response association between the number of pain sites and mental health problems has been found in adolescents [3], which is similar to the general population where multiple pains are associated with depression [19]. Most studies have examined the association between physical complaints and only a few psychosocial factors. There is a lack of a broader sense of exploration to determine the importance of relevant associating factors.

Anxiety/depressive disorders have been found in about 30% of patients presenting physical complaints in primary health care, and 38% in psychiatric clinics [19]. Low recognition of psychiatric disorders among adolescents in primary care has been shown [20], which is a matter of concern. Emotional disorders were most frequently identified and were significantly associated with high levels and intensity of physical symptoms [20]. The high reports of physical complaints in relation to mental health problems, and the limited recognition of psychiatric disorders by the general practitioner, warrant further investigation and focus on the current topic.

Few studies have examined ethnic/cultural differences in physical pain and the association with psychosocial factors [1,21]. Indigenous peoples have historically experienced several psychosocial traumas through harsh assimilation processes resulting in loss of ethnic identity, native language, land and traditional living conditions [22]. Lifetime PTSD has been found associated with pain in rural American Indians [23]. Pain studies on Sami, the indigenous population in Scandinavia, or other Arctic indigenous adolescent groups has not been done to our knowledge. A recent study has shown that Sami adolescents are not reporting more mental health problems than their majority Norwegian peers [24]. The attachment to harsh natural environments and hard physical work such as hunting, and primary industries like fishing and reindeer herding, may have influenced the Arctic indigenous peoples awareness of the body and perception of pain differently from their non-indigenous counterparts.

The aim of the study was first to explore the prevalence of widespread musculoskeletal pain in indigenous and non-indigenous Arctic adolescents. Second, to examine the association with physical-, psychosocial factors, and mental health problems in a hierarchical model to determine the importance of the factors. Third, to examine the influence of pain related functional impairment on this association.

## Methods

### Study design

The Norwegian Arctic Adolescent Health Study [25] was conducted among 10th graders (15–16 year olds) in all junior high schools in the three northernmost counties in Norway, in 2003–2005. The questionnaires were administered in classroom settings, monitored by project staff, and completed during two school hours. Few students used more than 45–60 minutes. Students who were not present in class during the questionnaire administration completed the questionnaire at a later date. The students and their parents were given written information about the study, and the students provided written consent. The data collection was conducted and funded by a joint collaboration between the Centre for Sami Health Research at the University of Tromsoe and the Norwegian Institute of Public Health. The Regional Medical Ethical Committee, the Norwegian Data Inspectorate, and the school authorities approved the study.

### Sample

A total number of 4,881 of 5,877 (RR: 83%) invited students accepted to participate, of whom 50.1% were female and 49.9% were male. 10% (450 of 4449) of the sample were indigenous Sami. In the non-Sami group 64 adolescents reported having other nationalities, thus consisting mainly of majority Norwegians.

### Variables

#### Physical factors

*Musculoskeletal pain* was measured by “yes/no” answers to the question: “*During the last 12 months have you several times been troubled by pain in the head, neck/shoulder, arms/legs/knees, abdominal or back?*” Abdominal pain was excluded on the basis of not necessarily originating from the abdominal muscles, and the potential bias of menstrual pain in females. Headache was included based on its frequent co-existence with musculoskeletal pain. Tension-type headache, the most common form of headache, and musculoskeletal pain have shared mechanisms and risk factors [26]. Headache, as a complaint, is much more common than migraine in adolescents (5-10% prevalence) [27-29]. The four pain sites included were handled as a discrete variable ranging from 0–4 pain sites.

*Pain related functional impairment*: Participants were asked if the pain had resulted in reduced activity during leisure time (yes/no). In the analyses for pain related functional impairment, those reporting functional impairment just due to abdominal pain were excluded (N = 26).

*Sedentary activity* was measured by the question: “After school hours: How many hours per school day (Monday to Friday) do you spend in front of TV, video, and/or PC?”; up to one hour (1), 1–2 (2), 3–5 (3), or >5 hours (4).

*Physical activity* was measured by the question: *“How many hours per week do you spend on physical activity, to an extent that make you sweat and/or out of breath”*; 0, 1–2, 3–4, 5–7, 8–10, or ≥11 hours per week. Physical activity was recoded into four groups, 0 (0), 1–4 (1), 5–7 (2) and ≥8 (3) hours per week [30].

*Self-rated health (SRH)* was measured by the question: *“How is your health right now?”* with four possible options. They were dichotomized into “not good/not so good” (0) and “good/very good” (1).

*Physical injury:* The participants were asked if they had experienced a serious illness or injury during the last year (yes/no), and were asked to describe their illness/injury. There were few reports of illness/injury of serious nature and the number of chronic illnesses reported was too low for statistical analysis. The number of physical injuries, mostly extremity injuries and some concussions, were 42.6% (N = 136) of the total responses.

#### Psychosocial and mental health factors

Mental health was examined by *anxiety/depression* symptoms measured by the Hopkins Symptom Checklist 10-item version (HSCL-10*)*[31], and by *mental health help seeking behavior* during the previous year (yes/no). The HSCL-10 (α = 0.86) measures symptoms of anxiety/depression in the previous week. Psychometrics has been empirically validated, also among subjects ages 16–24 and for Sami and non-Sami subjects in this study [24,32], with a cut-off of 1.85 of the sum score indicating a presence of emotional distress.

*Resilience* was measured by a 5-item version of the 10-item *General perceived self-efficacy scale*[33], with higher scores indicating higher resilience. In the 5-item version (α = 0.77) questions 1, 2, 4, 7 and 9 were used, scored on a four-point Likert scale from “completely wrong” (1) to “completely right” (4).

*Parental involvement* was measured by a 4-item version of the *Parental Involvement Scale* (α = 0.78) by Alsaker et. al. [34]. Based on the questions: *“My parents know where I am at and what I do in the weekend,” “my parents know where I am and what I do on weekdays,” “my parents know who I spend my leisure time with”* and *“my parents like the friends I spend time with.”*

*Parental support* (α = 0.88) was measured by the following five statements: *“I feel attached to my family,” ”my family takes me seriously,” “my family values my opinions,” ”I mean a lot to my family”* and *“I can count on my family when I need help.”*

*Peer support* (α = 0.84) was measured by the following four statements: *“I feel closely attached to my friends,” “my friends value my opinions,” “I can help/support my friends,”* and *“I can count on my friends when I need help”.*

*Parental involvement*, *parental* and *peer support* were all measured by a four-point Likert scale from “completely agree” (1) to “completely disagree” (4).

*School-related stress* (α = 0.66) was measured by the following experiences: *“Have you ever experienced any of the following:” “Heavy work pressure at school,” “heavy pressure from others to succeed/ do well at school,” “find it very difficult to concentrate in class”* and *“find it very difficult to understand the teacher when he/she is teaching?”* Responses were measured on a three-point Likert scale from “no” (1) to “yes, often” (3).

*Negative life events* were measured by the following 12 questions: *“Have you in the last 12 months had anyone of the following problems,” “conflict or fights with your parents,” “parental mental health problems,” “parental financial problems,” “parental drug problems” or “peer problems?”* Responses were measured on a four-point Likert scale from “no, never” (0), “yes, sometimes” (1), “several times” (2), to “very often” (3). Furthermore, respondents were asked, *“have you in the last 12 months experienced trouble being bullied at school/ on the way to school?”* with the following options: “never” (0), “sometimes” (1), “about once a week” (2), and “several times a week” (3). Also, *“Have you in the last 12 months been exposed to violence?”* with the following options of “never” (0), “yes, only by adolescents” (1), “yes, only by adults” (2), and “yes, by both adolescents and adults” (3). Lastly, respondents were asked, *“have you in the last 12 months experienced the following:” “parental unemployment or social care,” “serious illness or injury to yourself,” “serious disease or injury to someone close to you,” “death to someone close to you”* or *“sexual assault?”* The possible answers were yes (1) and no (0). All the variables above were dichotomized into any degree of exposure (1) and zero degree of exposure (0), resulting in range of negative life events from 0–12.

For *peer- and conduct problems* we used the Strengths and Difficulties Questionnaire (SDQ) [35] the conduct- (α = 0.47) and peer subscales (α = 0.52).

#### Socio-demographic factors

*Socioeconomic status:* Information was obtained about the participants parent’s occupation and was classified according to the International Standard Classification of Occupation, ISCO-88 [36], later reclassified into five categories based on the parent with the highest rated occupation. We controlled for the interaction with gender and family structure, which showed no difference. Parental work reported “unknown” was recoded into the missing group.

*Family income:* Adolescents reported their family’s economic situation compared to other families according to a 4-point scale from “not well off” (1) to “very well off” (4).

*Sami ethnicity* was measured by an assessment of Sami parentage and Sami language competence in parents, grandparents and the participants, and self-labeling. Participants having one or more of these factors present were classified as having Sami ethnicity [37].

### Data analysis

Pearson correlations were used to control for multicollinarity between explanatory factors by applying Cohen’s criteria [38].

Univariate analyses were carried out using Chi-square tests and one-way ANOVA, with post hoc comparison by Tukey. Hierarchical, backward, linear multiple regression analysis was used for analysis of significant univariate predictors. Stratified analyses for gender, ethnicity (Sami vs. non-Sami), and pain related functional impairment were carried out. All stratified regression analyses followed the subsequent steps: First, socio-demographic factors were entered, followed by physical factors, psychosocial factors, and mental health and conduct problems. The anxiety/depression variable was handled as a continuous variable in all multivariable analyses. The categorical variables were coded 1 for the presence of the phenomenon and 0 for its absence and for gender, females (0) and males (1).

Evaluation of the multivariable models explained variance was done by Cohen’s criteria: 2-13% is small, 13-26% is medium and ≥ 26% is large [38]. All analyses were conducted with the SPSS version 21 (IBM software). The statistical significance level were set to .01 due to the large number of participants, except for the Sami group where .05 was chosen due to lower N.

## Results

### Univariate findings

The prevalence of musculoskeletal pain and mental health problems were higher for females (Table 1), as was the number of pain sites (Table 2). For the total sample the prevalence of physical injury was 2.8% and the majority (88.5%) reported being in good/very good health (Table 1). Socioeconomic status of parents (data not shown) was not significantly associated with the number of musculoskeletal pain sites for neither gender nor ethnicity.

**Table 1:** Prevalence of musculoskeletal pain, self-rated health and mental health by ethnicity and gender (%)

	**Sami**				**Non-Sami**				**Total Ethnic difference**
**Variables**	**Males**	**Females**	**Total**	**Gender**	**Males**	**Females**	**Total**	**Gender diff.**	
	**N = 228**	**N = 222**	**N = 450**	**diff.**	**N = 1981**	**N = 2018**	**N = 3999**		
*Musculoskeletal pain:*									
Headache	42.7	65.6	54.0	21.79**	37.5	62.7	50.3	245.30**	1.97^p=.16^
Neck/shoulder pain	29.0	45.5	37.1	11.61**	27.2	42.5	34.9	98.02**	.71^p=.40^
Back pain	28.8	31.1	30.0	.16^p=.69^	30.8	38.0	34.4	21.73**	3.18^p=.08^
Arm/knee/leg pain	32.9	31.6	32.2	.03^p=.85^	29.5	33.6	31.6	7.39*	.06^p=.81^
*Self-rated health (SRH):*									
Good/very good SRH	89.2	86.6	88.0	.48^p=.49^	90.2	87.3	88.8	8.14*	.19^p=.67^
Physical injury	5.3	1.4^b^	3.3	4.20^p=.04^	3.3	2.4	2.9	2.61^p=.11^	.16^p=.69^
*Mental health:*									
Anxiety/depression^a^	7.6	32.0	19.6	40.43**	7.6	30.4	19.2	331.67**	.02^p=.88^
Help seeking behavior	1.8^c^	8.8	5.2	9.05*	3.5	9.5	6.5	55.69**	.85^p=.36^

Note: *p ≤ .01, **p ≤ .001.

^a^Hopkins Symptom Checklist-10, cut-off 1.85.

^b^Only 3 subjects. ^c^Only 4 subjects.

**Table 2:** Number of musculoskeletal pain sites by associated factors; gender, ethnicity, self-rated health, pain related functional impairment, and mental health problems (%, mean = M)

**Variables**	**Number of pain sites**						
	**N**	**0**	**1**	**2**	**3**	**4**	**χ**^**2**^**/F-ratio**
		**N = 1273**	**N = 1312**	**N = 944**	**N = 612**	**N = 410**	
*Gender (%):*							169.41**
Males	2286	34.4	30.8	19.1	9.5	6.3	
Females	2267	21.6	26.9	22.4	17.4	11.7	
*Ethnicity (%):*							
Sami	415	25.8	28.7	24.8	13.3	7.5	5.31^p=.26^
Non-Sami	3745	28.1	28.9	20.3	13.6	8.9	
*Self-rated health (SRH) (%):*							
Good/very good SRH	4500	94.7	91.7	88.2	81.6	72.1	198.94**
Physical injury	4553	1.5	2.1	3.6	3.9	5.9	29.68**
*Mental health (M/%):*							
Anxiety/depression (M)^a^	4533	1.27	1.35	1.52	1.75	1.92	225.02**
Males (≥1.85, %)	2275	4.0	4.7	9.2	14.3	21.8	82.02**
Females (≥1.85, %)	2263	14.4	16.9	32.1	46.6	63.4	297.09**
Sami (%)	414	7.5	14.4	23.3	29.1	45.2	29.41**
Non-Sami (%)	3737	7.5	9.9	21.8	36.1	51.1	473.01**
Help seeking behavior							
Males (%)	2259	2.5	2.0	4.6	5.5	5.7	13.96*
Females (%)	2225	5.4	4.3	8.5	14.4	20.6	77.34**
Sami (%)	406	2.9	1.7	4.9	7.3	14.3	10.10^p=.04^
Non-Sami (%)	3692	3.3	3.2	7.5	12.1	16.5	117.74**
*Pain related functional impairment (%):*							
*Yes:*	3345	10.2	31.4	42.6	53.2	60.7	260.19**
Anxiety/depression (≥1.85)^a^	1323	-^b^	12.8	31.0	42.3	58.4	149.42**
Help seeking behavior	1304	-^b^	3.5	8.6	11.4	20.3	45.76**
*No:*	3345	89.8	68.6	57.4	46.8	39.3	260.19**
Anxiety/depression (≥1.85)^a^	2003	11.1	10.0	15.4	27.7	31.1	105.17**
Help seeking behavior	1986	3.1	2.9	5.4	11.7	9.3	38.98**

Note: *p ≤ .01, **p ≤ .001.

^a^Hopkins Symptom Checklist-10, cut-off 1.85.

^b^Too low N in the zero pain brackets.

Sedentary activity was significantly associated with the number of pain sites (p ≤ .001), and physical activity for females only (p = .002). Post hoc analyses for sedentary activity in the total sample showed that those reporting >5 hours (M = 1.60) of sedentary activity reported significantly more pain sites than those reporting 1–2 hours (M = 1.37) and <1 hour (M = 1.37), but not 3–5 hours (M = 1.47). The post hoc comparisons showed that females reporting no physical activity (M = 1.96) reported significantly more pain sites than those reporting 1–4 (M = 1.67) and 5–7 hours (M = 1.64) of activity per week, but not those reporting ≥8 hours (M = 1.80).

We found a linear relationship between the increasing pain sites and the associating factors, with an increase in psychosocial (data not shown) and mental health problems, and a reduction in family income and resilience (data not shown). These factors, excluding peer support (p = .14), were significantly associated with musculoskeletal pain at the p ≤ .001 level. Family income was significant in females (p ≤ .001) only. Peer- (p = .18) and conduct problems (p = .07) were not significant in the Sami group.

There was an increasing level of mental health problems in those reporting more than one pain site (Table 2), particularly for those reporting four pain sites, where three times more females than males scored over the cut-off for anxiety/depression. The results concerning ethnicity showed the same tendencies for both ethnic groups (Table 2).

We found some minor ethnic differences in higher reports of back pain in non-Sami females (Table 1) and higher reports of mental health problems in the non-Sami reporting 3–4 pain sites (Table 2). There was no difference in the number of pain sites between the two ethnic groups (Table 2).

### Multivariable findings

Table 3 shows the multivariable analysis for the total sample and stratified by gender. In the final model, anxiety/depression, negative life events and school-related stress were the strongest factors associated with musculoskeletal pain, with anxiety/depression explaining most of the variance (3.0%) in the total sample. The final model accounted for a medium-high percentage of the explained variance of musculoskeletal pain in females, with medium-low explained variance for males. Gender, as the only significant socio-demographic factor, remained significant with females reporting more musculoskeletal pain. Physical injury and conduct problems were significant for males’ only, and sedentary activity only for the total sample.

**Table 3:** **Hierarchical, backward, linear multiple regression analysis between musculoskeletal pain, socio-demographic-, physical-, psychosocial factors, and mental health by gender (Final model: N**_**Total**_ **= 4290, N**_**Males**_ **= 2193, N**_**Females**_ **= 2163 )**

	**Musculoskeletal pain**		
	**Total sample**	**Males**	**Females**
Variables	β		
*Step 1:*			
Gender	-.19**	-	-
Family income	-.07**	-.04^p=.063^	-.11**
R^2^	.041**	.002^p=.063^	.012**
*Step 2:*			
Gender	-.19**	-	-
Family income	-.05*	-	-.08**
Physical activity	-	-	-
Sedentary activity	.08**	.07**	.08**
Self-rated health	-.18**	-.16**	-.20**
Physical injury	.08**	.09**	.06*
R^2^	.090**	.041**	.062**
*Step 3:*			
Gender	-.13**	-	-
Family income	-	-	-
Sedentary activity	.04*	-	-
Self-rated health	-.12**	-.11**	-.13**
Physical injury	.05**	.06*	-
Resilience	-	-	-
Parental involvement	-	-	-
Parental support	-	-	-
Peer problems	.05**	-	.07**
School-related stress	.20**	.20**	.23**
Negative life events	.18**	.17**	.20**
R^2^	.187**	.120**	.179**
*Step 4 (Final model):*			
Gender	-.07**	-	-
Sedentary activity	.04*	-	-
Self-rated health	-.08**	-.09**	-.08**
Physical injury	.06**	.07**	-
School-related stress	.12**	.13**	.13**
Negative life events	.13**	.11**	.15**
Conduct problems	.04*	.07**	-
Anxiety/depression^a^	.23**	.15**	.26**
Help seeking behavior	-	-	-
R^2^	.214**	.140**	.218**

Note: *p ≤ .01, **p ≤ .001.

^a^Hopkins Symptom Checklist-10.

We investigated the different types of negative life events and their association with musculoskeletal pain. When controlled for gender, and the events controlling for each other there were no standouts among the significant factors (β-values: .059-.126). The significant negative life events were: conflicts or fights with parents, parental financial problems, peer problems, bullying, exposure to violence, serious illness or injury, serious illness or injury to someone close, and sexual assault.

Table 4 shows the final multivariable regression model for Sami and non-Sami. For the Sami group only gender was significant in step 1 and 2, and in step 3 negative life events and school-related stress. Only anxiety/depression and negative life events were significant in the final model. As school-related stress did not reach statistical significance in the Sami group, we examined predictors for anxiety/depression in our sample. School-related stress (β_Sami_ = .24, p ≤ .001, β_non-Sami_ = .27, p ≤ .001) along with gender was the strongest predictor of anxiety/depression controlled for the other psychosocial factors in both ethnic groups.

**Table 4:** **Final model of linear multiple regression analysis**^**b **^**between musculoskeletal pain, socio-demographic-, physical-, psychosocial factors, and mental health by ethnicity (Final model: N**_**Sami**_ **= 413, N**_**Non-Sami**_ **= 3607)**

	**Musculoskeletal pain**	
	**Sami**	**Non-sami**
Variables	β	
*Step 4 (Final model):*		
Gender	-	-.07***
Sedentary activity	-	.04*
Self-rated health	-	-.07***
Physical injury	-	.07***
School-related stress	-	.13***
Negative life events	.13**	.12***
Conduct problems	-	.05**
HSCL-10^a^	.30***	.24***
Help seeking behavior	-	-
R^2^	.137***	.231***

Note: *p < .05. **p ≤ .01. ***p ≤ .001.

^a^Hopkins Symptom Checklist-10.

^b^Hierarchical, backward, linear multiple regression analysis following the same steps as the analyses in Table 3.

Table 5 and Table 2 show the importance of pain related functional impairment in relation to the associating factors, especially mental health problems. The final model for the associating factors had a much higher explained variance in the functional impairment group. Anxiety/depression was the strongest associating factor whether functional impairment or not.

**Table 5:** **Final model of linear multiple regression analysis**^**b **^**between musculoskeletal pain, socio-demographic-, physical-, psychosocial factors, and mental health by pain related functional impairment (N**_**Functional inpairment**_ **= 1230, N**_**No functional impairment**_ **= 1984)**

	**Musculoskeletal pain**	
	**Functional impairment**	**No functional impairment**
Variables	β	
*Step 4 (Final model):*		
Gender	-.09*	-
Sedentary activity	.07*	-
Self-rated health	-.07*	-
School-related stress	.09*	.10**
Negative life events	.08*	.14**
Conduct problems	.08*	-
Anxiety/depression^a^	.23**	.16**
Help seeking behavior	.08*	-
R^2^	.212**	.094**

Note: *p ≤ .01, **p ≤ .001.

^a^Hopkins Symptom Checklist-10.

^b^Hierarchical, backward, linear multiple regression analysis following the same steps as the analyses in Table 3.

## Discussion

### Main findings

The aims of this study were to examine the prevalence of musculoskeletal pain in Arctic adolescents with indigenous Sami and non-indigenous background, and to investigate the impact of a broad range of essential associating factors. The main findings were high reports of musculoskeletal pain and a strong association with psychosocial factors, especially in those reporting pain related functional impairment. There were no major ethnic differences between Sami and non-Sami in prevalence, however the pattern of predictors differed somewhat between the indigenous and non-indigenous group.

### Comparisons to previous studies

For the total sample anxiety/depression was the strongest factor, followed by negative life events and school-related stress. The only socio-demographic variable that remained significant in the multivariable analysis was gender, with females reporting more pains. Our main findings are in line with earlier findings [9-11] with emphasis on widespread musculoskeletal pain in adolescents.

The high prevalence of musculoskeletal pain, mental health problems, and their association is in accordance with earlier studies [2-4,9-11,16,39], with similar findings as a comparable survey done in other Norwegian counties [3,39]. A matter of concern was the high number of females with more than one pain site who reported above the clinical cut-off for anxiety/depression. Earlier studies [1,9,10,12,13] support that mental health problems are an important factor associated with musculoskeletal pain, particularly in females. The gender difference for musculoskeletal pain was predominantly explained by increased reports of psychosocial and mental health problems in females, but gender was still significant in the multivariable analysis.

We found no significant difference between the number of musculoskeletal pain sites and mental health problems in the indigenous Sami and their majority Norwegian peers. Overall we found the same trends with anxiety/depression as the dominant factor, but there were some differences in the multivariable analysis, even with a .05 significance level for the Sami group. It was surprising that gender, physical factors, and school-related stress did not make significant contributions to the model in the Sami group. There may be some underlying differences, but the most important sociocultural aspect of the findings is that the indigenous Sami are not worse off. The difference in power, due to the difference in participants, between the Sami and non-Sami group, and school-related stress mediating effect, may also explain the difference. In Norway, the indigenous Sami are well integrated into the Norwegian society and do not report more mental health problems [40]. Thus one should be careful in drawing similarities to other nations where socioeconomic and cultural differences between indigenous and non-indigenous groups are more pronounced.

The higher explained variance for musculoskeletal pain in the functional impairment group emphasizes the importance of pain related functional impairment. Mental help seeking behavior being statistically significant in the final model in the functional impairment group also supports this. Understandably pain related functional impairment should have a stronger relation to lower quality of life. The relation to mental health problems was particularly evident for those reporting more than one pain site.

We found school-related stress to be an important factor associated with musculoskeletal pain as Hjern et. al. [15], who found school environment important for pain. However, our questions were more specific on stress/pressure at school, as we assumed these types of complaints were related to musculoskeletal pain. School is an important arena in adolescence both socially and academically, and its importance as a potential stressful arena should not be neglected.

Negative life events was an important factor, supporting earlier studies [1,15,41]. Negative life events, not necessarily causing mental health problems, were also associated with musculoskeletal pain. There were no standouts among the negative life events. This supports a general perspective on negative life events and the recognition that several events might be of relevance. This would depend on the adolescents’ own subjectivity of distress concerning the events in question.

Conduct problems in males were significantly associated with musculoskeletal pain, supporting that there are some gender differences in associating factors for adolescents. Behavioral problems have been found as predictive for pain in males [10,12], and in both genders [13].

In general, the adolescents reported being in good health and reported a low prevalence of physical injury in spite of high reports of musculoskeletal pain. Self-rated health made a significant contribution to the final model for both genders, while physical injury only for males. The prevalence of physical injury might be low in our sample due to the respondents’ interpretation of level of seriousness in their injury, even though there were few reports of a serious nature. In a German self-reported sample, the number of respondents who had sports injuries during the previous year was 8% [42]. Excluding a somatic cause for the musculoskeletal pain was not possible in this study due to the nature of the survey.

The finding that sedentary activity was associated with musculoskeletal pain in both genders supports Paananen et al. who found it associated with high sitting time. Hoftun et. al. who found it significant only in females [11]. Sedentary activity had a stronger association to musculoskeletal pain compared to low physical activity, which we found significant in females in the univariate analysis. This is in contrast to Hoftun et al. who found it so in both genders [11] and to Paananen et al. who found high physical activity to be associated with musculoskeletal pain [9]. In regard to our post hoc results one can argue that both groups are at higher risk, but hypothesize that it is more likely due to mechanical factors, such as injuries, in highly active individuals.

Earlier studies have shown significant association with psychosomatic complaints and poor resilience, and poor parental affection [14,17], as our univariate findings support. However, resilience and parental factors lost their significance when stronger factors as anxiety/depression were included, but they may act as mediating factors.

### Study strengths and weaknesses

A strength of this study is that it incorporates several important psychosocial factors associated with musculoskeletal pain in an integrated model. Another strength is its multiethnic sample, and indigenous Sami adolescents were included from the same ethnic contexts as their majority Norwegian peers.

The study had a high participant rate and a high number of participants from all the junior high schools in North Norway. Though it is known that non-responders might be more troubled than responders, the missing data were few regarding each question. This suggests that the adolescents understood the questions well, which contributes to the internal validity of the study.

Due to its cross-sectional design, no causal direction of the association can be described. Psychosomatic problems are a two-way street and reverse causality is possible. As most large scale population-based studies this study relied on self-reports and are thus at risk for information bias. The lower internal consistencies of the SDQ-subscales are debatable, but these are well-used scales in epidemiological and clinical work.

The dependent pain question can be criticized. The specification of one year is wide and at risk for recall bias. More likely the answers represent a shorter time period of 3–6 months. The expression “several times” is, objectively, a vague specification of prevalence and is open for interpretation, but it indicates some regularity and seriousness of pain. Even so, when handling the number of pain sites as a discrete variable the importance of increasing pain sites association with psychosocial problems is emphasized. Because the coexistence of musculoskeletal pain from different locations is high, it is better handled as a continuum. Due to the nature of the survey we could not differentiate which of the pains had led to functional impairment.

### Clinical implications

This study emphasize that increasing musculoskeletal pain sites presented by adolescents increases the probability of comorbid psychosocial problems. Physicians meeting young people presenting multiple or recurrent musculoskeletal pain should therefore look for problems like anxiety and depression, school-related stress and negative life events, and consider pain related daily life functioning. The adolescents should be offered interventions for their problems.

## Conclusions

We found no significant difference in the prevalence of widespread pain between indigenous Sami and their majority Norwegian peers, but some differences in the multivariable analyses of associating factors. The most important sociocultural aspect of the findings is that the indigenous Sami are not worse off. We found a strong dose–response association between widespread musculoskeletal pain and psychosocial factors Anxiety/depression, negative life events, and school-related stress were the most important factors associated with musculoskeletal pain, especially in those reporting pain related functional impairment.

## Abbreviations

SDQ: Strengths and difficulties questionnaire; HSCL-10: Hopkins symptom checklist 10-item version; SRH: Self-rated health.

## Competing interests

The authors declare that they have no competing interests.

## Authors’ contributions

SK is the project manger of The Norwegian Arctic Adolescent Health Study, and responsible for design and data collection. In this study SK has contributed in obtaining funding, hypothesis, analysis and interpretation, critical revision of the article, and supervision. CE has contributed in conception and design of this study, analysis and interpretation, writing and critical revision of the article. The authors meet the authorship conditions. The authors read and approved the final manuscript.

## Pre-publication history

The pre-publication history for this paper can be accessed here:

http://www.biomedcentral.com/1471-2458/14/617/prepub
